# Acute Relaxation Response Induced by Tibetan Singing Bowl Sounds: A Randomized Controlled Trial

**DOI:** 10.3390/ejihpe13020024

**Published:** 2023-01-29

**Authors:** Cristobal Rio-Alamos, Rodrigo Montefusco-Siegmund, Toni Cañete, Joaquín Sotomayor, Alberto Fernandez-Teruel

**Affiliations:** 1 Faculty of Medicine, Department of Psychology, Austral University of Chile, Valdivia 5090000, Chile; 2 Human Cognitive Neurophysiology and Behavior Lab, Locomotor Apparatus and Rehabilitation Institute, Faculty of Medicine, Department of Kinesiology, Austral University of Chile, Valdivia 5090000, Chile; 3 Centro Interdisciplinario de Estudios del Sistema Nervioso, Austral University of Chile, Valdivia 5090000, Chile; 4 Medical Psychology Unit, Department of Psychiatry and Forensic Medicine, Institute of Neurosciences, Autonomous University of Barcelona, 08193 Barcelona, Spain

**Keywords:** anxiety, music therapy, Tibetan singing bowl, heart rate variability, relaxation response

## Abstract

The prevalence of anxiety has increased dramatically due to COVID-19, so effective preventive interventions are welcome. The main objective of our study was to compare the acute relaxation response (RR) induced by Tibetan singing bowl (TSB) sound-based treatment against progressive muscle relaxation (PMR) and a control waiting list group (CWL) in a single treatment session in an adult nonclinical anxious population. In this cross-sectional randomized control trial, 50 participants selected based on high state anxiety were randomly assigned to one of the experimental groups. Pre/post self-reported anxiety, electroencephalographic activity (EEG), and heart rate variability (HRV) were recorded at baseline (T1), minute 15 (T2), minute 30 (T3), and minute 45 (T4). The TSB group showed significant reductions in alpha power (from T2 to T4) and increased HRV (from T3 to T4) compared with the PMR and CWL groups. Moreover, TSB and PMR both showed significant reductions in self-reported anxiety compared with CWL, with this effect being more evident in the TSB group. We concluded that a single session of TSB treatment was able to induce a more evident psychological/physiological relaxation response compared with PMR and CWL. TSB could be a relevant acute intervention in stressful situations or crisis intervention and while waiting for conventional interventions.

## 1. Introduction

Anxiety is considered one of the major mental health disorders and represents a highly relevant societal problem. It is usually defined as a complex phenomenon characterized by a state of high arousal and negative valence, often leading to enhanced vigilance in the absence of an immediate threatening stimulus [1,2,3,4]. The prevalence of anxiety has been estimated at 7.3% of the worldwide population [5]. However, the coronavirus disease 2019 (COVID-19) pandemic significantly impacted mental health conditions, raising anxiety prevalence over three times, reaching a 25% prevalence for the general population [6,7]. In Chile, the prevalence for risk of anxiety has been estimated to be 39.2% of the population. In this context, effective therapeutic strategies aiming to reduce anxiety/stress responses are of great relevance. For this reason, despite advances in pharmacological treatments and psychotherapy, innovative and preventive approaches are still required, mainly because it is known that a significant proportion of patients do not respond adequately to pharmacotherapy, which can be associated with undesirable side effects (e.g., sexual dysfunction, excessive perspiration, drowsiness, and weight gain) [8,9,10,11].

Regarding effective complementary interventions, it has been proposed that treatments that are able to induce a relaxation response (RR) would be valuable tools for anxious patients [12,13,14,15]. Herbert Benson, the first to describe RR, defined it as the physiological counterpart of the stress response [12,13,16]. RR is known as a set of integrated physiological mechanisms and adjustments that can be elicited through mind–body techniques, aiming to restore the activity imbalance between the sympathetic and parasympathetic nervous systems (SNS and PNS, respectively) [12,15,16]. Thus, it has been proposed that RR inhibits SNS activity, counteracting the stress response by activating the PNS [15,16]. Hence, a more active PNS appears to be a key feature for anxiety/stress modulation. Previous studies lend support to the above contention, having shown several PNS-like responses elicited by RR techniques. These include decreased oxygen consumption, lower heart rate and blood pressure, and reductions in HPA-related hormone levels, self-reported anxiety, and neuroelectric alpha power band (8–12 Hz), as well as increased heart rate variability (HRV), among other psychological/physiological responses [14,15,17,18,19,20,21,22].

Although RR can be elicited through many different techniques (e.g., autogenic training, meditation, mindfulness-based programs, etc.), Jacobson’s Progressive Muscle Relaxation (PMR) is considered a kind of theoretical cornerstone for RR acquisition [15,21,23,24]. The effects of PMR have been thoroughly described elsewhere, but some relevant outcomes reported include better control of anxiety [25,26,27,28,29], reductions in perceived anxiety/stress [17,30,31], and reductions in cortisol and salivary alpha-amylase levels [17,27,28,29]. However, despite the validity acquired by PMR treatment on parasympathetic activation, some limitations have been pointed out, especially when used clinically: (i) PMR requires expert direction; (ii) it demands an active role by the patient; and (iii) it is “not so fun”, thereby decreasing treatment adherence.

On the other hand, one promising complementary treatment is to use the relaxing sounds of Tibetan singing bowl (TSB) sound-based treatment. TSB consists of metal alloys used originally by Tibetan monks for meditative ceremonies [17,31,32,33,34,35]. The sound consists of a base tone that merges with a series of overtones, producing a continuous relaxing sound. Evidence suggests that TSB can induce improvements in distress, anxiety, depression, fatigue, and tension, as well as improvements in blood pressure, heart rate, respiratory rate, peripheral capillary oxygen saturation, cutaneous conductance, and alpha power measured by electroencephalography (EEG) [17,31,32,34]. Interestingly, a study compared monochord sound treatment, which shares the same principles as TSB treatment (a base tone merged with overtones producing a relaxing continuous sound), with PMR on self-reported anxiety and EEG. The results showed that both monochord sound treatment and PMR were able to induce RR, with the former being more effective [31]. Moreover, TSB has also been used in preoperative anxiety conditions, showing improvements in hormonal responses and self-reported anxiety measurements when compared with a control group. Specifically, TSB was able to induce: (i) reductions in self-reported anxiety; (ii) reductions in alpha-amylase levels; (iii) increases in high frequency of HRV; and (iv) increases in theta power band activity (3.5–7.5 Hz) and reductions in beta power band activity [17,31,32].

We were interested in examining the efficacy of TSB using a cross-sectional design as a starting point, evaluating the potential benefits of TSB used in an occasionally anxious (i.e., nonclinical) population. The results of acute nonpharmacological anxiety-related interventions (e.g., music, aromatherapy, massage, and acupuncture) reported by systematic reviews have shown significant positive effects of music as an effective intervention [36,37,38]. Similar to our study, a cross-sectional study showed that music and PMR were able to reduce anxiety after a one-day treatment session [39]. Thus, the aim of our study was to compare the potential acute RR effects induced by TSB treatment against PMR and a control waiting list (CWL) over a single treatment session. To achieve this aim, self-reported anxiety, HRV, and neuroelectrical activity (EEG) were obtained from all participants. We hypothesized that both TSB and PMR would be able to induce RR effects, with the results being more evident in the TSB group.

## 2. Materials and Methods

### 2.1. Design and Participants

**Figure 1:**
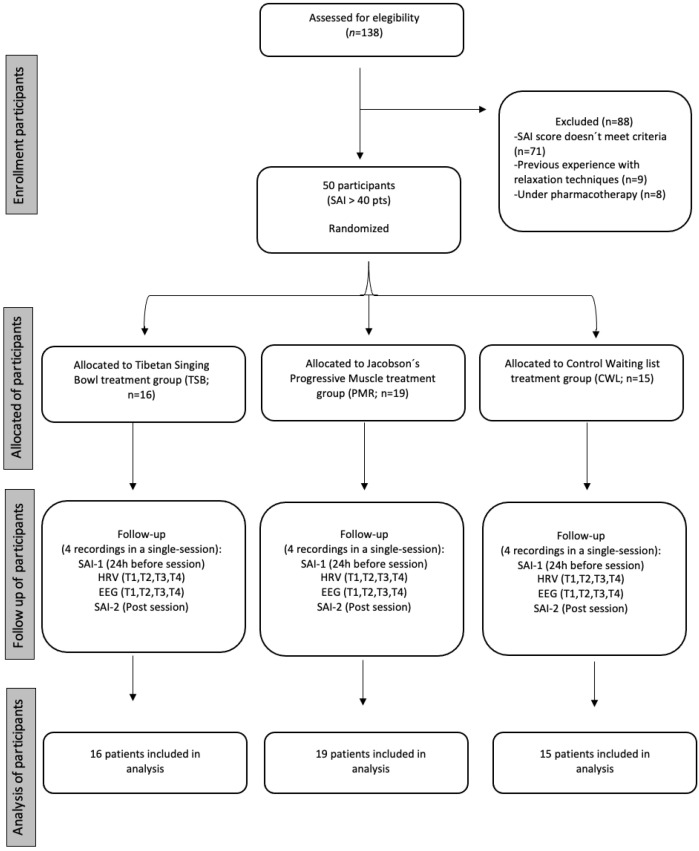
Study flow diagram.

This single-blind prospective randomized control trial was conducted during periods of remission of the quarantine imposed by local governments regarding the COVID-19 pandemic. All sanitary safeguards were applied. The study protocol followed the Consolidated Standards of Reporting Trials (CONSORT) criteria, as shown in Figure 1.

For selection criteria, all participants signed a written consent form and completed the Spanish version of Spielberger State Anxiety Inventory (SAI) and a questionnaire about their physical condition. Selected participants (>40 in SAI) were randomly assigned to one of three experimental groups: TSB, PMR, or CWL. A final sample of 50 subjects was recruited (TSB = 16, PMR = 19, CWL = 15; *n*/per group). The exclusion criteria were previous experience with TSB or PMR, regular practice of relaxation techniques, and being under current psychiatric pharmacotherapy. A total of 50 participants were recruited (26 ♂ and 24 ♀: TSB (*n* = 16): 9 ♂/7 ♀; PMR (*n* = 19): 10 ♂/9 ♀; CWL (*n* = 15): 7 ♂/8 ♀). Regarding their education level, 30 participants (60%) were current university students, and 20 participants (40%) had finished their university studies and were currently working. No significant differences were found (TSB = 26.2 ± 1.5; PMR = 26.3 ± 1; CWL = 25.9 ± 1).

### 2.2. Outcome Measures

#### 2.2.1. Heart Rate Variability (HRV)

HRV recordings were obtained using a Polar H10 heart rate sensor device located at the chest and connected via Bluetooth to EliteHRV software. Once recordings were finished, data were transferred to a computer, and analyses were performed using Kubios HRV software (Kubios HRV, version 3.4.3) [40]. HRV artifacts were removed before data analyses using an automatic beat correction algorithm and the threshold-based beat correction algorithm provided by Kubios software [40]. Data were analyzed considering two HRV dimensions (time and frequency) related to vagal tone activity. The HRV time domain variables were: (i) the root mean square of successive heartbeat interval differences (RMSSD) and (ii) the percentage of successive heartbeat intervals that differed by more than 50 milliseconds (pNN50) [22,41]. The HRV frequency domain included: (i) VLF: very low frequency, ranging from 0 to 0.4 Hz; (ii) LF: low frequency from 0.04 to 0.15 Hz; and (iii) HF: high frequency from 0.15 to 0.4 Hz. Here we focused on HF, as it is considered a measure of parasympathetic activity only [41].

#### 2.2.2. Electroencephalography (EEG)

**Figure 2:**
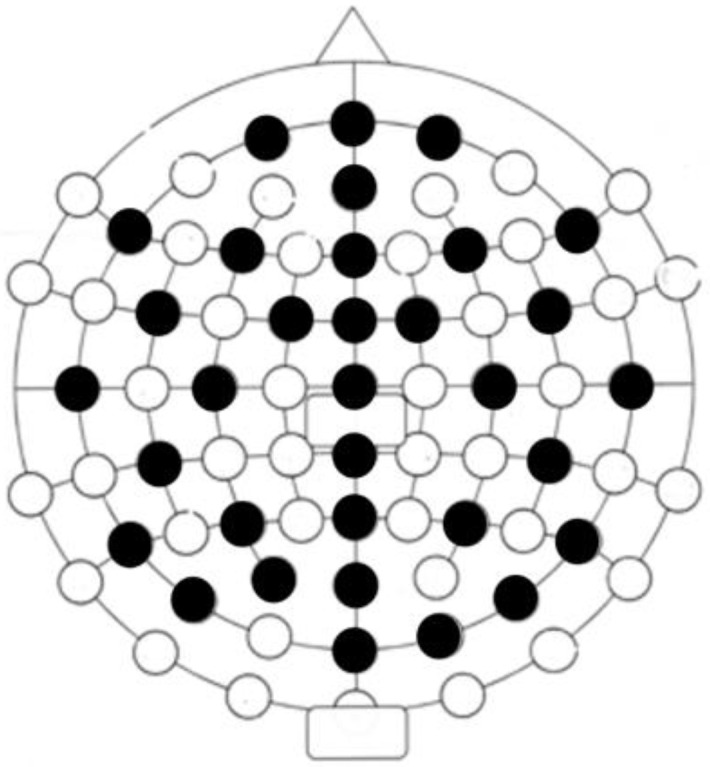
EEG channel allocation with a 10–20 system map (active channels in black circles).

EEG measurements were recorded and saved for off-line analysis using a 32-channel Emotiv Epoc Flex gel-based sensor device connected via USB to Emotiv Pro Software (version 2.6.4) (see Figure 2 for channel locations). The EEG signals were digitized at a sampling rate of 128 Hz. Each electrode impedance was kept below 5 kΩ. The EEG signals were re-referenced off-line to the algebraic mean of the two earlobe electrodes. EEG analyses were performed using Matlab (Mathworks) and the open-source fieldtrip Matlab toolbox (http://www.ru.nl/fcdonders/fieldtrip/, (accessed on 1 November 2022)) [42]. The spectral content of the frequency bands was calculated using a Hanning taper before Fourier transformation. The mean spectral power was calculated for standard frequency bands: theta (3.5–7.5 Hz), alpha (8–12 Hz), and beta (12.5–29.5 Hz).

#### 2.2.3. Spielberg’s State Anxiety Inventory (SAI)

The Spanish version of Spielberg’s State Anxiety Inventory (SAI) is a subscale of the State Trait Anxiety Inventory (STAI), which assesses the current state of anxiety. It consists of 20 items, with a total score ranging from 20 (almost never) to 80 (almost always). A higher score indicates greater anxiety, and a cut-off point of 39–40 has been suggested to detect clinically significant symptoms [31,43].

### 2.3. Interventions

#### 2.3.1. Tibetan Singing Bowl (TSB)

TSB sessions were performed live by an expert (i.e., a trained music therapist). Four types of TSB were used. For accurate identification of the frequency sound of each TSB, a professional recording session was conducted in an anechoic chamber (Acoustic Institute of the Austral University of Chile). Recordings were analyzed with Audacity software (version 3.0.2.0) (see Table 1 for TSB specifications). Each TSB session was played the same, whereby higher sound frequencies were first delivered, followed by the progressive appearance of lower sound frequencies. The TSB sound was elicited by striking the TSBs and by rubbing them with a wooden mallet.

#### 2.3.2. Jacobson’s Progressive Muscle Relaxation (PMR)

PMR instructions were provided using a Bose Bluetooth Soundlink Revolve portable black speaker. The PMR session was recorded and edited in a professional recording studio in such a way that it was synchronized with the silent periods of the TSB group. Participants were instructed about tensing and relaxing the body muscles with a bottom-up procedure (i.e., beginning with the feet, followed by the legs, abdomen, chest, hands, shoulders, neck, face, and head) [44].

#### 2.3.3. Control Waiting List (CWL)

Participants in the CWL group remained silent throughout the session, and primary and secondary outcomes were recorded exactly the same as in the TSB and PMR groups.

### 2.4. Procedure

All experimental procedures were performed between 10:00 and 13:00. The experimental room was kept at 22 ± 2 °C, and all recordings were obtained individually. Once they arrived, the participants were asked to sit in a comfortable armchair for HRV-EEG preparation. The SAI-1 score was obtained as both a selection criterion and basal self-reported anxiety (pre-session). Once the devices were set up properly, the experimental session began with a continuous eyes-closed EEG-HRV recording for 50 min. Within the experimental session, four [4] silent periods were included for data analysis: from 0 to 5 min (T1, baseline); from 15 to 20 min (T2); from 30 to 35 min (T3); and from 45 to 50 min (T4, post-session). Once the HRV-EEG devices were removed, the participants were asked to complete SAI-2 (post-session).

Ethical approval was provided by the Ethical Committee of Bioethics on Human Research of the Austral University of Chile (Ethical Committee approval *n*º 0041/19) in November 2019. The clinical protocol was reviewed and approved by ISRCTN, with clinical trial registration number ISRCTN5397685.

### 2.5. Statistical Analyses

Statistical analyses were performed using the Statistical Package for the Social Sciences (SPSS, version 17, IBM Corp.) and Matlab (Mathworks) software. Appropriate repeated measures ANOVA with “SAI-1/SAI-2” and “T1 to T4 EEG/HRV” as the within-subject factor and “treatment group” as the between-subject factor was performed. One-way ANOVA was applied to all dependent variables at each time interval (EEG/HRV T1-T4). The SAI-R was obtained by calculating the difference between SAI-1 and SAI-2. Reductions in alpha activity were obtained by calculating the difference between T1 and T2, T1 to T3, and T1 to T4. Then, one-way ANOVA was applied to the SAI-R and alpha reduction computed variables. Post-hoc Duncan’s multiple range test was applied to all dependent variables following significant ANOVA effects. Pearson’s correlation coefficients were performed among the main variables. Obliquely rotated factor analysis (direct oblimin) was performed with the main variables recorded. The significance level was set at *p* ≤ 0.05. All variables were normally distributed.

## 3. Results

**Figure 3:**
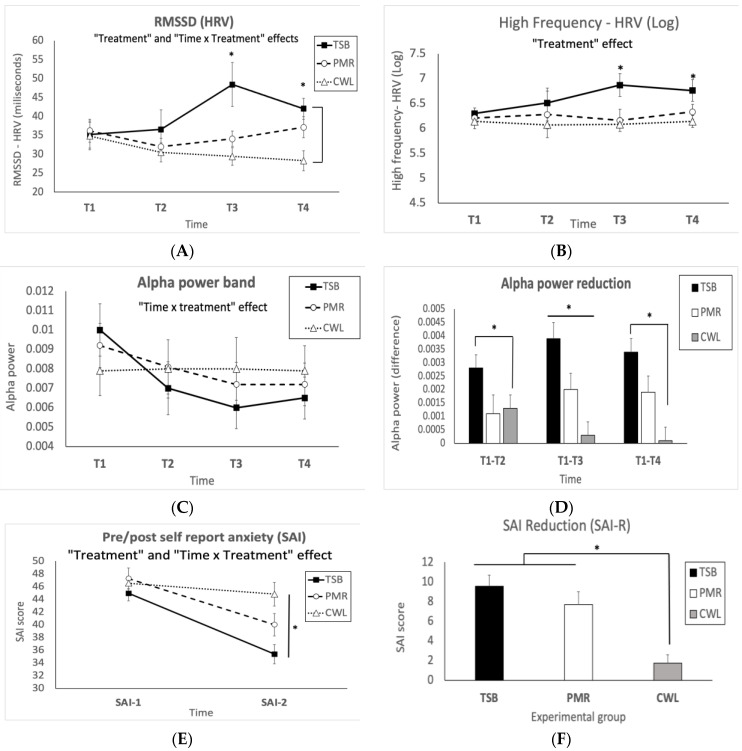
Mean ± SEM of (**A**) Root mean square of successive heartbeat interval differences (RMSSD) trajectory indicating “treatment” and “treatment x time” effects, as the TSB group showed higher RMSSD than the PMR and CWL groups, especially in T3 and T4. (**B**) Trajectory of the HRV high-frequency(log) indicating a “treatment” effect, as overall, significant differences between TSB and PMR/CWL were observed, especially at T3 and T4. (**C**) Occipital alpha band power along the session indicating a “time x treatment effect”, as the TSB group showed a significant reduction in alpha power (T3) in comparison with the PMR/CWL groups (**D**) Reduction of alpha power between T1 and T2, T1 and T3, and T1 and T4, indicating a significant reduction of occipital alpha band activity between TSB and CWL across the whole session and against PMR at T3. (**E**) SAI-1 and SAI-2 scores showing SAI reductions in TSB and PMR against CWL. (**F**) SAI reductions (SAI-R), indicating a greater SAI reduction in both TSB and PMR against CWL; this effect was more evident in the TSB group. Total sample *n* = 50 (TSB:16/PMR:19/CWL:15); * *p* < 0.05 between groups indicated (Duncan’s multiple range tests following significant ANOVA effects). Group symbols: TSB, Tibetan singing bowl; PMR, progressive muscle relaxation; CWL, control waiting list.

**Figure 4:**
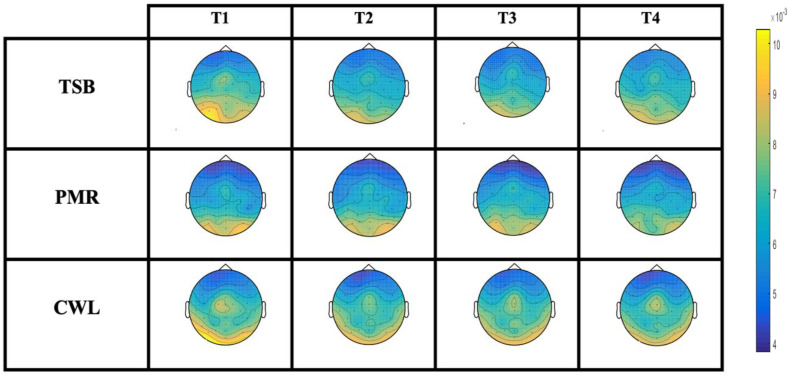
EEG brain map activity for the alpha band over the experimental session. Group symbols: TSB, Tibetan singing bowl; PMR, progressive muscle relaxation; CWL, control waiting list. Total sample *n* = 50 (TSB:16/PMR:19/CWL:15).

No differences were observed between the groups in all variables at T1. The main statistical effects between the experimental groups among all dependent variables are described in Table 2 and Figure 3 and Figure 4.

Pearson’s correlation analyses between the main variables (EEG/HRV T1–T4; SAI-1, SAI-2, SAI-R, and alpha reduction) showed moderate negative associations between SAI-2 and all alpha activity reduction (r = −0.30 to −0.37, the lower the SAI-2, the higher the alpha reduction). SAI-2 also showed moderate negative associations with RMSSD at T3 and T4 (r = −0.039 to −0.41). The same negative associations were observed between SAI-2 and HRV-HF(log) (r = −0.029 to −0.033, the lower the SAI-2, the higher the RMSSD/HRV-HF(log)). High positive associations between RMSSD and pNN50/HRV-HF(log) were found from T1 to T4 (r = 0.40 to 0.89). As no associations were found between the HRV and EEG variables, we performed obliquely rotated factor analysis (direct oblimin) with the main theoretical and significant variables. The results identified two independent factors (see Table 3).

Figure 3A–F mean and SEM of all main dependent variable effects.

## 4. Discussion

The primary purpose of this study was to compare the potential acute relaxation response (RR) induced by Tibetan singing bowl (TSB) sound-based treatment with Jacobson’s progressive muscle relaxation (PMR) and a control waiting list group (CWL) on heart rate variability (HRV), alpha power band (EEG), and self-reported anxiety in a young adult nonclinical anxious population.

The main results of our study are: (i) The TSB group showed differences in both HRV time/frequency domain related to parasympathetic activation. Specifically, the TSB group showed higher RMSSD and HRV high frequency (HRV-HF(log)) at T3 and T4 compared with the PMR and CWL groups; (ii) the TSB group showed significant reductions in the alpha power band on EEG compared with the PMR and CWL groups (from T2 to T4); and (iii) the TSB and PMR groups showed significant reductions in self-reported anxiety (SAI) compared with the CWL group, with this reduction being more evident in the TSB group.

To the best of our knowledge, this is the first time that TSB treatment has been compared with PMR and a CWL in which HRV, EEG, and self-reported anxiety were recorded altogether. Previous studies have compared TSB effects against either a control group or a PMR group, as well as between naïve versus experienced meditators, but none have included both PMR and CWL groups [15,16,21,31,34]. We found that both TSB and PMR treatments were able to promote a psychological and/or physiological RR in comparison with the CWL group, with these effects being more evident in the TSB group.

It has been proposed that one key feature of RR acquisition is a more active parasympathetic nervous system that is able to counterbalance sympathetic activity [15,16,45,46,47]. HRV has been considered a valid physiologic marker related to the ability to better cope with stressful situations. HRV can measure overall nervous system activity by observing changes in the heart rate cycle, as the excitement of the sinus node controlled by the autonomic nervous system is responsible for heart rate [47,48,49,50]. HRV parameters (i.e., RMSSD, pNN50, and HRV-HF) have been shown to be consistently associated with anxiety disorders (e.g., panic disorder, general anxiety disorder, and post-traumatic stress disorder, among others), and increases in these HRV parameters were promoted by pharmacotherapy, psychotherapy, or relaxation interventions [22,41,48]. Our results are in line with previous data, showing that TSB and PMR treatment were able to increase HRV parameters, which is usually associated with higher vagal tone or parasympathetic activity (i.e., RMSSD, pNN50, and HRV-HF(log)) [22,41,50,51,52]. Specifically, the TSB group showed a higher RMSSD at T3 (vs. PMR) and at T3 and T4 (vs. CWL) (Figure 3A), as well as an overall increase in pNN50 vs. the PMR/CWL groups (Table 2). Moreover, higher HRV-HF(log) at T3 and T4 (vs. PMR and CWL) was also observed (Figure 3D). PMR also showed higher RMSSD vs. CWL at T4, which is a finding that was expected. The HRV trajectory might be interpreted as progressive activation of the parasympathetic nervous system as the experimental session was running. The peak of the HRV RR was at minute 30 (T3), a time point at which EEG outcomes were also more evident. Interestingly, our HRV trajectories resemble those observed in previous studies in which a single TSB treatment session of 20 or 40 min was able to promote RR indexed by HRV parameters and self-reported mood, also showing the peak of relaxation at T3 (30 min) [53,54]. These results might indicate that the time of exposure seems to be relevant for the induced RR effects; however, the impact of the sound frequency of the TSB at this moment (T3) cannot be ruled out. Future studies are needed to address this question.

Regarding the EEG results, the TSB group showed a significant reduction in alpha band activity from T2 to T4 (vs. CWL) and at T3 (vs. PMR) (Figure 3E,F). Divergent and inconclusive EEG outcomes related to anxious/relaxed states have been reported [14,31,55,56]. However, two main streams of conclusions have been validated above others. First, the “theta hypothesis” proposes that relaxed states and mental concentration (such as meditative states) correlate with increases in frontal midline theta rhythms, something that has also been observed with anxiolytic drugs [56,57,58,59]. On the other hand, the “alpha hypothesis” proposes that alpha oscillations are enhanced in anxious individuals, particularly in anxiogenic environments, being interpreted as a sign of greater hypervigilance and readiness of the alpha system for information processing [3,19,20,60,61,62,63], a hypothesis supported by Gray’s theory of anxiety [3,64]. Previous studies have shown that alpha oscillations are positively correlated with trait/state anxiety [19]. It has also been demonstrated that higher alpha power band activity in anxious subjects increases even more after an alarming/startling stimulus [19,55,65]. Thus, whereas previous studies have shown that anxious subjects increase alpha power after an alarming/stressful event, along the same line, we observed the opposite effect as a consequence of RR acquisition induced by the TSB intervention. That is, subjects with high state anxiety showed decreased alpha activity elicited by TSB sound-based treatment as an index of relaxation in which environmental alertness seemed to cease to be so relevant. Thus, a possibility is that TSB treatment desynchronized alpha activity in such a way that subjects were more “disconnected” from the environment (i.e., reduction of hyperalertness toward the environment). This suggested relaxing effect is consistent with the experimenter’s observational report, noting that TSB participants were significantly more restful and drowsy at the end of the session, something that was clearly reflected in post-session self-reported anxiety (SAI-2).

Regarding self-reported anxiety, the TSB and PMR groups showed significant reductions in SAI-2 compared with the CWL group, with this effect being more evident in the TSB group. Interestingly, the TSB group’s SAI-2 scores were reduced and located below the cut-off point suggested to detect clinically significant symptoms (<40), indicating a more profound subjective relaxing state, which was expected considering the pleasant nature of this intervention as well as previous findings [17,31,32,34]. PMR also reduced SAI-2 scores but in a less evident manner. This could be because PMR needs more active and focused attention, especially when patients are unfamiliar with the technique. On the other hand, TSB is a more passive and pleasant intervention that provides an interesting tool for anxiety management.

One interesting feature observed in our study is that the HRV-EEG trajectories from T1 to T4 describe a very similar and consistent progression (Figure 3A–D). In this regard, it was tempting to hypothesize associations between HRV and EEG, which was not observed. Pearson’s correlation analysis showed moderate positive associations between SAI-R and RMSSD (i.e., the higher the SAI reduction, the higher the RMSSD), as well as between SAI-R and EEG alpha reduction (at T3) (i.e., the higher the SAI-R, the higher the alpha activity reduction). This is in line with previous findings [22,47,50,52] and with the expected results (i.e., TSB’s anxiolytic effects); however, no associations were found between HRV and EEG. This could indicate that two independent mechanisms might have been involved in the observed RR. In fact, when factorial analysis was performed, two independent dimensions were identified. The first dimension grouped SAI measurements (i.e., SAI-2 and SAI-R) with RMSSD, and the second grouped SAI (as in the first dimension) with alpha activity reduction (see Table 3). However, the possibility of associations between HRV and EEG variables cannot be ruled out. The neurovisceral integration model proposes that cardiac vagal tone, as reflected by HRV, is associated with prefrontal attentional control, indicating the ability to self-regulate in a more flexible manner under stress situations [45,46,66,67]. HRV is suggested to be a good index that reflects those neural mechanisms of central nervous system/autonomic nervous system integration [47,67]. Specifically, the central autonomic network, composed of several subcortical structures, innervates the heart, first through preganglionic sympathetic and parasympathetic neurons and finally through the stellate ganglia and vagus nerve. Therefore, the interplay between sympathetic and parasympathetic outputs would be responsible for HRV. The idea is that reductions in vagally mediated cardiovascular control disinhibits sympatho-excitatory influences that are usually under chronic inhibition. Thus, our HRV results might be considered an activation of vagal tone, restoring the chronic sympathetic inhibition and promoting a better balance between the sympathetic and parasympathetic nervous systems, and through this balance, eliciting a more relaxing sensation. On the other hand, as EEG measures the electrical activity generated by large groups of neurons, it is not possible to determine specific subcortical functional activity through this technique. Thus, EEG is not the proper way to test the neurovisceral hypothesis. However, it is important to highlight the “SAI-EEG” and “SAI-HRV” associations because they indicate that anxious/relaxed states are somehow related to both neural and cardiac dimensions.

In conclusion, our results show that TSB and PMR were able to promote RR (psychological and/or physiological) over a single treatment session, with this effect being more evident in the TSB group. Specifically, TSB induced increases in HRV parameters related to parasympathetic activation, as well as reductions in alpha band activity, which can be seen as a possible sign of reduced environmental hyperalertness. Moreover, both TSB and PMR reduced self-reported anxiety scores, with this effect being more evident in the TSB group. Our results are in line with previous reports that showed similar TSB relaxation-inducing effects from a single treatment session [53,54]. We believe that this acute effect is a relevant feature of TSB treatment and complements the findings reported by TSB longitudinal studies [17,31,32,34]. As TSB is a passive, relaxing, and non-effort treatment for the patient, it might be an excellent intervention for the anxious environment as well as a complementary treatment for anxiety crisis. However, future studies are still necessary in order to evaluate the extent of long-lasting vs. short-term effects, as well as whether TSB’s effects are due to “time session” (i.e., if the TSB-inducing effects depend more on the time of TSB exposure) or the “frequency tone” (i.e., the frequency of the TSB used in the procedure and at each particular interval).

## Figures and Tables

**Table 1:** Description of the diameter and frequency of tone and overtones of the four Tibetan singing bowls used.

Tibetan Singing Bowl *n*º-(Diameter)	Tone Frequency (Hz)	1º Overtone (Hz)	2º Overtone (Hz)	3º Overtone (Hz)	4º Overtone (Hz)
1-(23 cm)	99	263	465	737	971
2-(15 cm)	219	603	1115	1713	2515
3-(12 cm)	465	1185	2224	3471	4865
4-(8 cm)	927	1879	2401	4437	-

**Table 2:** Mean and SEM of all dependent variables with their statistical effects.

	Mean ± (SEM)			ANOVA Repeated Measures	One-Way ANOVA
Dependent variable	TSB (*n* = 16)	PMR (*n* = 19)	CWL (*n* = 15)	“Effect” (F) * *p*	(F) * *p*
RMSSD (T1)	35.2 (3.55)	36.19 (3.04)	34.81 (3.63)	“Treatment” (3.6) * 0.04 “Time × treatment” (4.2) * 0.001 Figure 3A	
RMSSD (T2)	36.54 (5.17)	32 (2.20)	30.5 (2.50)		
RMSSD (T3)	48.4 (5.81)	34.07 (2.04)	29.4 (2.30)		(8.0) * 0.001
RMSSD (T4)	42.02 (2.78)	37.12 (2.76)	28.3 (2.65)		(6.5) * 0.003
pNN50 (T1)	14.7 (2.69)	12.05 (2.33)	19.90 (3.4)	“Time × treatment” (5.2) * 0.001	
pNN50 (T2)	16.5 (3.7)	8.84 (2.30)	10.8 (2.2)		
pNN50 (T3)	29.05 (5.55)	10.2 (2.73)	16.74 (4.77)		
pNN50 (T4)	16.94 (2.06)	16.16 (3.04)	14.62 (4.4)		
HRV-HF(log) (T1)	6.3 (0.11)	6.21 (0.07)	6.14 (0.15)	“Treatment” (7.4) * 0.002 Figure 3B	
HRV-HF(log) (T2)	6.51 (0.3)	6.28 (0.47)	6.07 (0.08)		
HRV-HF(log) (T3)	6.87 (0.23)	6.16 (0.22)	6.08 (0.14)		(8.5) * 0.001
HRV-HF(log) (T4)	6.76 (0.22)	6.33 (0.15)	6.14 (0.12)		(8.3) * 0.001
Alpha (T1)	0.01 (0.0013)	0.009 (0.0011)	0.007 (0.0012)	“Time × treatment” (4.9) * 0.001 Figure 3C	
Alpha (T2)	0.007 (0.0013)	0.008 (0.0013)	0.008 (0.0014)		
Alpha (T3)	0.006 (0.001)	0.007 (0.0011)	0.008 (0.0016)		
Alpha (T3)	0.006 (0.001)	0.007 (0.0011)	0.008 (0.0012)		
Alpha (T1–T2)	0.0028 (0.0005)	0.0011 (0.0007)	0.0013 (0.0008)		(3.9) * 0.03
Alpha (T1–T3)	0.0039 (0.0006)	0.002 (0.0006)	0.0003 (0.0006)		(8.0) * 0.001
Alpha (T1–T4)	0.0034 (0.0005)	0.0019 (0.0006)	0.0001 (0.0005)		(8.4) * 0.001 Figure 3D
SAI-1	44.94 (1.1)	47.3 (1.7)	46.3 (0.82)	“Treatment” (9.2) * 0.001 “Time × treatment” (11.61) * 0.001 Figure 3E	
SAI-2	35.37 (0.27)	40.0 (0.78)	44.8 (0.80)		(45.9) * 0.001
SAI-R	9.56 (1.1)	7.68 (1.3)	1.73 (0.85)		(11.6) * 0.001 Figure 3F

**Table 3:** Factor analysis. Loadings ≥ 0.40 are shown.

	*Factor 1*	*Factor 2*
*SAI-1*	0.53	0.42
*SAI-2*	−0.47	0.81
*Alpha (T1)*	-	-
*Alpha reduction (T1–T3)*	-	−0.82
*RMSSD (T1)*	0.83	-
*RMSSD (T3)*	0.86	-
*% of variance (cumulative)*	37.18	59.57
*Correlation*	−0.13	
*N sample =*	27	

## Data Availability

The datasets generated during and/or analyzed during the study are available from the corresponding author on reasonable request.
